# GenoBaits Cassava35K: high-resolution multi-SNP arrays for genetic analysis and molecular breeding using targeted sequencing and liquid chip technology

**DOI:** 10.1093/hr/uhae305

**Published:** 2024-10-24

**Authors:** Chaochao Li, Xiaoxue Ye, Zhongxin Jin, Kaisen Huo, Jiangxiang Ma, Weiwei Tie, Zehong Ding, Yongfeng Zhou, Wei Hu

**Affiliations:** National Key Laboratory for Tropical Crop Breeding, Hainan Key Laboratory for Biosafety Monitoring and Molecular Breeding in Off-Season Reproduction Regions, Key Laboratory of Biology and Genetic Resources of Tropical Crops, Coconut Research Institute, Institute of Tropical Bioscience and Biotechnology, Sanya Research Institute, Chinese Academy of Tropical Agricultural Sciences, 4 Xueyuan Road, Haikou 571101, China; Hainan Key Laboratory for Protection and Utilization of Tropical Bioresources, Hainan Institute for Tropical Agricultural Resources, Chinese Academy of Tropical Agricultural Sciences, 4 Xueyuan Road, Haikou 571101, China; National Key Laboratory for Tropical Crop Breeding, Hainan Key Laboratory for Biosafety Monitoring and Molecular Breeding in Off-Season Reproduction Regions, Key Laboratory of Biology and Genetic Resources of Tropical Crops, Coconut Research Institute, Institute of Tropical Bioscience and Biotechnology, Sanya Research Institute, Chinese Academy of Tropical Agricultural Sciences, 4 Xueyuan Road, Haikou 571101, China; Hainan Key Laboratory for Protection and Utilization of Tropical Bioresources, Hainan Institute for Tropical Agricultural Resources, Chinese Academy of Tropical Agricultural Sciences, 4 Xueyuan Road, Haikou 571101, China; National Key Laboratory for Tropical Crop Breeding, Hainan Key Laboratory for Biosafety Monitoring and Molecular Breeding in Off-Season Reproduction Regions, Key Laboratory of Biology and Genetic Resources of Tropical Crops, Coconut Research Institute, Institute of Tropical Bioscience and Biotechnology, Sanya Research Institute, Chinese Academy of Tropical Agricultural Sciences, 4 Xueyuan Road, Haikou 571101, China; Hainan Key Laboratory for Protection and Utilization of Tropical Bioresources, Hainan Institute for Tropical Agricultural Resources, Chinese Academy of Tropical Agricultural Sciences, 4 Xueyuan Road, Haikou 571101, China; National Key Laboratory for Tropical Crop Breeding, Hainan Key Laboratory for Biosafety Monitoring and Molecular Breeding in Off-Season Reproduction Regions, Key Laboratory of Biology and Genetic Resources of Tropical Crops, Coconut Research Institute, Institute of Tropical Bioscience and Biotechnology, Sanya Research Institute, Chinese Academy of Tropical Agricultural Sciences, 4 Xueyuan Road, Haikou 571101, China; Hainan Key Laboratory for Protection and Utilization of Tropical Bioresources, Hainan Institute for Tropical Agricultural Resources, Chinese Academy of Tropical Agricultural Sciences, 4 Xueyuan Road, Haikou 571101, China; National Key Laboratory for Tropical Crop Breeding, Hainan Key Laboratory for Biosafety Monitoring and Molecular Breeding in Off-Season Reproduction Regions, Key Laboratory of Biology and Genetic Resources of Tropical Crops, Coconut Research Institute, Institute of Tropical Bioscience and Biotechnology, Sanya Research Institute, Chinese Academy of Tropical Agricultural Sciences, 4 Xueyuan Road, Haikou 571101, China; Hainan Key Laboratory for Protection and Utilization of Tropical Bioresources, Hainan Institute for Tropical Agricultural Resources, Chinese Academy of Tropical Agricultural Sciences, 4 Xueyuan Road, Haikou 571101, China; National Key Laboratory for Tropical Crop Breeding, Hainan Key Laboratory for Biosafety Monitoring and Molecular Breeding in Off-Season Reproduction Regions, Key Laboratory of Biology and Genetic Resources of Tropical Crops, Coconut Research Institute, Institute of Tropical Bioscience and Biotechnology, Sanya Research Institute, Chinese Academy of Tropical Agricultural Sciences, 4 Xueyuan Road, Haikou 571101, China; Hainan Key Laboratory for Protection and Utilization of Tropical Bioresources, Hainan Institute for Tropical Agricultural Resources, Chinese Academy of Tropical Agricultural Sciences, 4 Xueyuan Road, Haikou 571101, China; National Key Laboratory for Tropical Crop Breeding, Hainan Key Laboratory for Biosafety Monitoring and Molecular Breeding in Off-Season Reproduction Regions, Key Laboratory of Biology and Genetic Resources of Tropical Crops, Coconut Research Institute, Institute of Tropical Bioscience and Biotechnology, Sanya Research Institute, Chinese Academy of Tropical Agricultural Sciences, 4 Xueyuan Road, Haikou 571101, China; Hainan Key Laboratory for Protection and Utilization of Tropical Bioresources, Hainan Institute for Tropical Agricultural Resources, Chinese Academy of Tropical Agricultural Sciences, 4 Xueyuan Road, Haikou 571101, China; National Key Laboratory of Tropical Crop Breeding, Shenzhen Branch, Guangdong Laboratory of Lingnan Modern Agriculture, Key Laboratory of Synthetic Biology, Ministry of Agriculture and Rural Affairs, Agricultural Genomics Institute at Shenzhen, Chinese Academy of Agricultural Sciences, 7 Pengfei Road, Shenzhen 518124, China; National Key Laboratory for Tropical Crop Breeding, Hainan Key Laboratory for Biosafety Monitoring and Molecular Breeding in Off-Season Reproduction Regions, Key Laboratory of Biology and Genetic Resources of Tropical Crops, Coconut Research Institute, Institute of Tropical Bioscience and Biotechnology, Sanya Research Institute, Chinese Academy of Tropical Agricultural Sciences, 4 Xueyuan Road, Haikou 571101, China; Hainan Key Laboratory for Protection and Utilization of Tropical Bioresources, Hainan Institute for Tropical Agricultural Resources, Chinese Academy of Tropical Agricultural Sciences, 4 Xueyuan Road, Haikou 571101, China

Dear Editor,

Cassava (*Manihot esculenta*) is a plant of the genus *Manihot* in the family Euphorbiaceae, which is extensively cultivated in over 100 countries and serves as a food source for ~1 billion people worldwide [1, 2]. The storage roots of the cassava plant contain a high starch content, comprising 16.6%–34% of the fresh storage roots [3]. Cassava is a C3–C4 plant with distinctive attributes, including resilience to drought and barrenness, and high productivity [4, 5]. Currently, the pivotal genes linked to cassava’s agronomic characteristics have been extensively investigated [6–8]. However, the high cost of genotype detection technology has resulted in a slow rate of development of cassava genome–related research in low-income countries. Therefore, the development of a low-cost, high-throughput, flexible, and efficient genotyping platform is crucial for cassava functional genome research and genomic selection breeding.

The single-nucleotide polymorphism (SNP) array is a well-established high-throughput genotyping tool that identifies bases at each target locus and analyzes the sample genotype by interacting the sample DNA with probes on the SNP array. It offers advantages such as high throughput, accuracy, reproducibility, and relatively low cost. SNP arrays are frequently employed in agricultural research for a range of applications, including population genetics analysis, genetic mapping, genome-wide association analysis, and variety protection [9]. Nevertheless, to date, no research has been conducted on the subject of SNP arrays in cassava.

GenoBaits is a targeted genotyping sequencing technology based on liquid-phase probe hybridization [10]. To select suitable target sites on the SC205 reference genome, we collected variation datasets from 377 cassava samples [7], filtered them to obtain 116 317 SNPs, and optimized the probe design to select 35 369 SNPs as background sites. From the previously published cassava agronomic trait–related regions, we filtered 917 trait-related SNPs, including 24 agronomic traits and 35 storage root metabolites [7, 8]. After evaluation, we selected 586 loci as foreground sites. Probe design was performed for 35 955 SNPs (Fig. 1A). Subsequently, 203 cassava samples were subjected to examination using GenoBaits Cassava35K, of which 202 samples exhibited locus detection rates exceeding 90%.

The SNP density map indicates that the target sites are distributed in the high gene density regions of the chromosome (Fig. 1B). Specifically, 28.72% of the sites are located in the intergenic region, 27.96% in the intronic region, and 16.59% in the exonic region (Fig. 1C). The gap length between neighboring SNPs were counted, and they conform to a normal distribution with an average GAP length of 17.59 Kb (Fig. 1D). To compare the consistency of the GenoBaits Cassava35K and resequencing data, we selected the SNP data of 197 identical samples in the two variant datasets. We analyzed the correlation between genomic nucleotide polymorphisms (π) of the same genome segment between GenoBaits Cassava35K array data and resequencing (Reseq) data; the π calculated from two datasets are significantly positively correlated (Fig. 1E). Among the 197 identical samples, there are 182 cultivars and 15 landraces. We calculated the fixation index (FST) between cultivars and landraces using the SNP array and resequencing data. The high FST regions on chromosomes are consistent between the two datasets, for example, in chr12:2.63–3.33 Mb, high FST was identified in both datasets (Fig. 1F).

**Figure 1:**
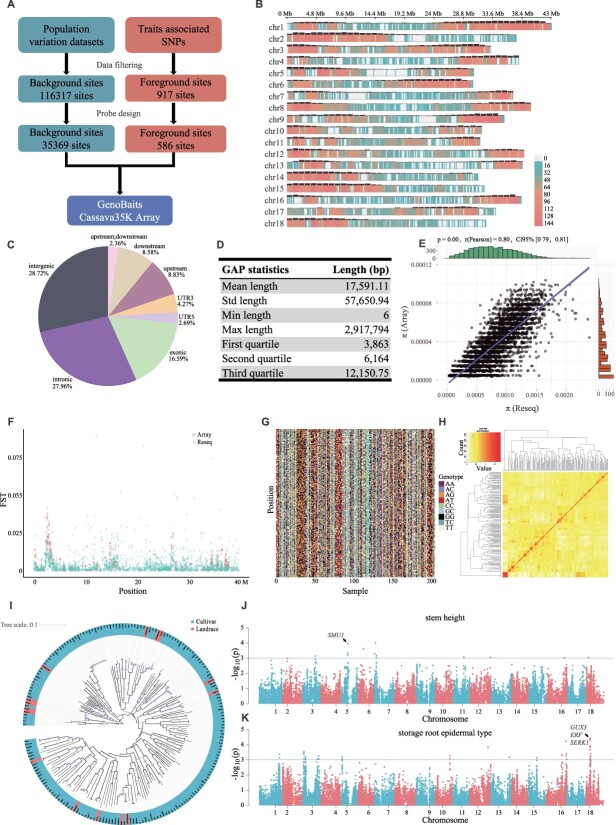
Development, evaluation, and application of the GenoBaits Cassava35K array. (A) Development of the GenoBaits Cassava35K array for cassava. (B) The density of SNP markers and gene density within a 1 Mb window of the cassava genome. (C) The distribution proportion of GenoBaits Cassava35K SNP site on the genome. (D) The length and distribution frequency of gaps between adjacent SNPs. (E) Correlation between the genome nucleotide polymorphism (π) calculated from the GenoBaits Cassava35K array and resequencing data using the same genome segment. (F) The FST between cultivars and landraces was calculated using the GenoBaits Cassava35K array and resequencing data from 197 identical samples. (G) SNP fingerprint map of cassava, which was constructed using 203 evenly distributed and highly polymorphic loci on the genome. (H) Genetic relationship matrix of cassava accessions. (I) Phylogenetic tree constructed using the GenoBaits Cassava35K array. (J, K) Gene localization related to stem height and storage root epidermal type in cassava accessions, with genes related to stem height and storage root epidermal type.

We comprehensively evaluated the application value of the GenoBaits Cassava35K from the perspectives of variety conservation, population genetic analysis, and genome-wide association study (GWAS). In order to construct a cassava DNA fingerprint, the cassava SNP dataset contained 203 representative cassava accessions. By selecting highly polymorphic sites (sites in the top 10% of nucleotide polymorphisms on each chromosome), we screened 203 sites distributed on the whole genome and constructed a cassava SNP fingerprint, which can be used for varietal identification and protection (Fig. 1G). The phylogenetic tree, principal components analysis (PCA), and population structure analysis were constructed using 197 identical samples from the SNP array dataset and resequencing data. Unluckily, as with the results of the previous resequencing data, PCA and population structure analyses were not able to discriminate well between cassava cultivars and landraces, which may be related to the domestication history of cassava. In the kinship matrix, we observed several cultivars with very close relatives, which should be avoided in subsequent crossbreeding to avoid inbreeding depression (Fig. 1H). To compare the differences between the phylogenetic trees constructed from the two datasets, we used the NyeSimilarity method to compare their topological consistency (Fig. 1I). The results showed that the trees from both datasets were 96.04% topologically similar. We collected stem height and storage root epidermal type from 203 samples. By comparing mixed linear model (MLM), general linear model (GLM), compressed mixed linear model (CMLM), and FarmCPU models, we performed GWAS analysis using the FarmCPU model. We localized two genes associated with stem height *Sc05g011620* (Transposon Ty3-G Gag-Pol polyprotein, *TY3B-G*) and *Sc05g011630* (Supporter of mec-8 and unc-52 protein homolog 1, *SMU1*), *SMU1* appears to be involved in the splicing of specific pre-messenger RNAs that affect multiple aspects of development (Fig. 1J). The genes related to storage root epidermal type are mainly located on chromosomes 3, 16, and 18. The genes on chromosome 18 have the highest significance, and most of them are related to cell growth, development, and hormone signaling. Among them, Sc18g008960 (UDP-glucuronate:xylan alpha-glucuronosyltransferase 3, *GUX3*), Sc18g009840 (Ethylene-responsive transcription factor, *ERF*), and Sc18g009860 (Somatic embryogenesis receptor kinase 1, *SERK1*) have been reported to be key genes that may regulate storage root empirical type (Fig. 1K).

In this study, we developed a cassava SNP array, GenoBaits Cassava35K, based on liquid probe hybridization–based targeted genotype testing technology. We validated the application of GenoBaits Cassava35K in genotyping, variety conservation, population structure analysis, and GWAS through full-scale testing of the dataset. Our SNP array outperforms other genotyping methods in terms of cost-effectiveness and flexibility. The field of cassava functional genomics is rapidly advancing, and GenoBaits Cassava35K must continually expand the number of functional regions associated with key traits in cassava to enhance its utility for functional research and molecular breeding.

## Data Availability

The GenoBaits Cassava35K dataset and supplementary files have been uploaded to GitHub (https://github.com/Licc900/GenoBaits_Cassava35K).
